# Real Time Monitoring of NADPH Concentrations in *Corynebacterium glutamicum* and *Escherichia coli* via the Genetically Encoded Sensor mBFP

**DOI:** 10.3389/fmicb.2018.02564

**Published:** 2018-10-24

**Authors:** Oliver Goldbeck, Alexander W. Eck, Gerd M. Seibold

**Affiliations:** ^1^Institute of Microbiology and Biotechnology, Ulm University, Ulm, Germany; ^2^Institute for Biochemistry, University of Cologne, Cologne, Germany

**Keywords:** *Corynebacterium glutamicum*, *Escherichia coli*, NADPH, redox state, biosensor, short chain dehydrogenase

## Abstract

Analyses of intracellular NADPH concentrations are prerequisites for the design of microbial production strains and process optimization. mBFP was described as metagenomics derived, blue fluorescent protein showing NADPH-dependent fluorescence. Characterization of mBFP showed a high specificity for binding of NADPH (*K*_D_ 0.64 mM) and no binding of NADH, the protein exclusively amplified fluorescence of NADPH. mBFP catalyzed the NADPH-dependent reduction of benzaldehyde and further aldehydes, which fits to its classification as short chain dehydrogenase. For *in vivo* NADPH analyses a codon-optimized gene for mBFP was introduced into *Corynebacterium glutamicum* WT and the phosphoglucoisomerase-deficient strain *C. glutamicum* Δ*pgi*, which accumulates high levels of NADPH. For determination of intracellular NADPH concentrations by mBFP a calibration method with permeabilized cells was developed. By this means an increase of intracellular NADPH concentrations within seconds after the addition of glucose to nutrient-starved cells of both *C. glutamicum* WT and *C. glutamicum* Δ*pgi* was observed; as expected the internal NADPH concentration was significantly higher for *C. glutamicum* Δ*pgi* (0.31 mM) when compared to *C. glutamicum* WT (0.19 mM). Addition of paraquat to *E. coli* cells carrying mBFP led as expected to an immediate decrease of intracellular NADPH concentrations, showing the versatile use of mBFP as intracellular sensor.

## Introduction

The redox state of cells is represented by the ratio of the internal concentrations of the pyridine nucleotides NADH and NADPH and their corresponding oxidized forms NAD^+^ and NADP^+^ (Blacker et al., 2014). These ubiquitous cofactors are present in cells only in catalytic amounts, therefore efficient recycling is required for the maintenance of viability and antioxidant protection (Krömer et al., 2008; Agledal et al., 2010; Mailloux et al., 2011). Intracellular availability of NADPH is of special relevance for microbial production processes like the biosynthesis of medicinal compounds, alcohols, biopolymers, and especially amino acids like L-lysine and L-valine (Weckbecker and Hummel, 2004; Sanchez et al., 2006; Bartek et al., 2010; Chemler et al., 2010; Kocharin et al., 2013; Lindner et al., 2013; Zhao et al., 2017). *Escherichia coli* and *Corynebacterium glutamicum*, two workhorses for the production of commodity chemicals (Becker and Wittmann, 2015), maintain their NADPH supply mainly via the pentose phosphate pathway (PPP) and the tricarboxylic acid cycle (Marx et al., 1996; Sauer et al., 2004; Spaans et al., 2015). To increase productivity and yields of microbial production strains current metabolic engineering strategies aim at an improved NADPH availability such as the redirection of the metabolism toward the PPP or introduction of transhydrogenases (Kabus et al., 2007; Lindner et al., 2013; Wang et al., 2017). Analyses of intracellular NADPH concentrations and their changes provide the basis for rational strain design and optimization and serve also for the detailed understanding of physiological roles of NADPH (Blacker et al., 2014; Ng et al., 2015; Liu and Wang, 2017). However, research is limited due to the technically challenging and labor-intensive analysis of internal NADPH concentrations (Lu et al., 2018).

Genetically encoded biosensors provide non-invasive optical readouts for internal concentrations of many different metabolites (Bolbat and Schultz, 2017; Lin et al., 2017). These sensors allow high throughput analyses of intracellular metabolite levels in microorganisms as a novel approach for strain selection and can also be employed to monitor metabolite levels in the course of cultivations (Schallmey et al., 2014; Eggeling et al., 2015; Bolbat and Schultz, 2017). For latter application transcription factor (TF) based sensors and the commonly used auto-fluorescent GFP derivatives come along with the drawback of poor dynamic behaviors (Liu et al., 2015; Liu and Wang, 2017), caused by their oxygen dependency and slow response times. For analysis of intracellular NADPH availability, only few biosensors have been described: The [2Fe-2S]-cluster containing transcriptional regulator SoxR of *E. coli*, was recently used for the design of a NADPH biosensor. Depending on the redox status of SoxR expression of the fluorescent protein eYFP is activated (Siedler et al., 2014). SoxR remains reduced and inactive as long as NADPH-dependent reductases are not limited in their substrate. Lack of NADPH leads to activation of SoxR and thus expression of eYFP. The SoxR-dependent NADPH biosensor was successfully applied to screen *E. coli* strains harboring gene bank derived variants of an NADPH consuming alcohol dehydrogenase (Siedler et al., 2014). A second NADPH sensor named iNAP recently described by Tao et al. (2017) reduces the probability of false positive readouts due to its ratiometric signal. The synthetic iNAP sensor consists of circularly permutated eYFP fused to the NADH binding domain of Rex from *Thermus aquaticus* (Zhao et al., 2015), of which the nucleotide binding pocket was subsequently mutated for NADPH-specificity by switching conserved residues (Tao et al., 2017). The iNap sensor offers a fast, non-TF-based response to changes in the NADPH/NADP^+^ ratio in various types of cells (Zhao et al., 2016; Tao et al., 2017).

The protein mBFP was recently described as NADPH-dependent, metagenomics derived, blue fluorescent protein: upon binding of NADPH mBFP amplifies the intrinsic fluorescence of NADPH in an oxygen independent manner (Hwang et al., 2012), and produces more fluorescence when supplied with more NADPH (Ng et al., 2015). Oxygen independent fluorescent proteins such as mBFP, whose fluorescent properties rely on the binding of the metabolite of interest, are generally ideal candidates for *in vivo* analytics of metabolite concentrations in the course of cultivation (Lin et al., 2017). The NADPH dependent fluorescence of mBFP were exploited to screen a series of *E. coli* strains for improved NADPH regeneration (Ng et al., 2015). Based on its amino acid sequence mBFP is classified as short chain dehydrogenase (SDR), however, no experiments were conducted in this direction so far. The analysis of fast variations of intracellular NADPH concentrations by the use of genetically encoded biosensors was to our knowledge hitherto not reported.

In this study, we characterized NADPH binding, fluorescence properties, and enzymatic activities of purified mBFP. Based on this knowledge of its biochemical parameters, we optimized application of this highly specific NADPH sensor for the *in vivo* analysis of fast changes in intracellular NADPH concentrations in both *C. glutamicum* and *E. coli*. The results of these experiments demonstrate that mBFP is a versatile tool for the quantitative determination of internal NADPH concentrations and their fast alterations in bacteria.

## Materials and Methods

### Bacterial Strains, Plasmids, and Culture Conditions

Bacterial strains and plasmids used in this study are listed in Table 1. Pre-cultures of *E. coli* and *C. glutamicum* were carried out in 2xTY medium in baffled Erlenmeyer flasks on a rotary shaker (130 rpm) at 30 and 37°C, respectively. CgXII was used as minimal medium for *C. glutamicum* (Eggeling and Bott, 2005) with 10 g L^-1^ glucose as carbon source. Strains carrying plasmids were cultivated in the presence of kanamycin (50 μg/mL) and IPTG (1 mM) for *mBFP* expression. Growth of *E. coli* and of *C. glutamicum* was followed by measuring the optical density (OD) at 600 nm in an Ultrospec 2100 pro spectrophotometer (GE Healthcare Life Sciences GmbH, Freiburg, Germany).

**Table 1:** Strains and plasmids used in this study.

Strains and plasmids	Relevant properties and applications	Sources and reference
**Strains**		
*E. coli* DH5α	F^-^ *thi-1 endA1 hsdR17* (r^-^ m^-^) *sup*E44 *Δlac*U169 (φ80l*acZ*ΔM15) *recA1 gyr*A96 r*el*A1 F^-^^-^ *ilvG rfb*-50 *rph*-1	Hanahan, 1983
*E. coli* BL21 (DE3)	F^-^ *ompT gal dcm lon hsdS*B(rB–mB–) [*malB* + ]K-12(S)	Studier and Moffatt, 1986
*C. glutamicum* ATCC13032	Wild type	American Type Culture Collection
*C. glutamicum*Δ*pgi*	In-frame deletion of *pgi* gene (cg0973) of *C. glutamicum* ATCC13032	Lindner et al., 2013
**Plasmids**		
pEKEx2	Ptac, *lacI^*q*^*, Km^r^	Eikmanns et al., 1991
pCN_mBFP	Expression plasmid carrying mBFP under constitutive promoter	Ng et al., 2015
pEKEx2_mBFPopt	Expression plasmid carrying the codon-optimized gene for mBFP under the control of the IPTG inducible Ptac promoter	This work

### Construction of Plasmid pEKEx2_mBFPopt

For plasmid construction, transformatiotransformation and plasmid isolation from *E. coli* DH5α standard cloning and molecular biology procedures were employed (Sambrook et al., 2001), transformation of *C. glutamicum* strains by electroporation was conducted as described (Tauch et al., 2002). Recombinant strains were selected using 2xTY-agar plates containing kanamycin (50 μg/mL). Eurofins MWG (Ebersberg, Germany) carried out synthesis of the mBFPopt gene fragment for a *C. glutamicum* codon-optimized mBFP gene (Supplementary Table S1). The optimized gene was amplified via PCR using the primers mBFPopt_fw and mBFPopt_rev (Supplementary Table S1) resulting in a 780 bp amplicon, which was subsequently digested with the restriction endonucleases *SacI* and *SalI*. Ligation of the 766 bp fragment into *SacI* and *SalI* linearized pEKEx2 (8,130 bp) resulted in the final construct pEKEx2_mBFPopt (8,896 bp). The plasmid pEKEx2_mBFPopt was controlled by restriction digestions and DNA sequencing (MWG Eurofins).

### Fluorescence Analysis

Fluorescence measurements were carried out in black 96-well plates (Sarstedt, Nümbrecht, Germany) in a TECAN infinite M200 plate reader (Tecan Group, Männedorf, Switzerland) equipped with an injection module. NADPH, NADH and mBFP holoenzyme fluorescence was recorded at an emission of 451 nm and excitation at 390 nm. For fluorescence kinetic measurements cells were harvested after overnight cultivation by centrifugation (4,000 rpm, 8 min, 4°C), washed twice with PBS (137 mM NaCl, 10 mM Na_2_HPO_4_, 1.8 mM NaH_2_PO_4_, pH 7.4) and suspended in PBS to an OD_600_ of 1. Fluorescence kinetic measurements were performed using read intervals of 1–2 s and automatic injection of substrate (100 mM glucose final concentration) at indicated time points. For kinetic measurements of the effects of paraquat first glucose (final concentration 100 mM) was added after 30 s of pre-incubation to cells suspended in PBS and then at the indicated time point paraquat (8–16 mM final concentration) was added. For analysis of mBFP fluorescence during cultivation of *C. glutamicum*, cultures were sampled after 2 h of cultivation, and the OD_600_ of the sample set to 1 with CgXII minimal medium before the fluorescence was measured in the plate reader.

Fluorescence microscopy was performed with an Axio Observer Z1 microscope (Zeiss, Oberkochen, Germany) using Zen software. For the visualization of mBFP fluorescence, cells were cultivated and treated as described above for kinetic measurements; 2% (w/v) glucose (final concentration) were added immediately before immobilization on agarose pads (1% w/v of agarose in PBS). For detection of mBFP fluorescence the Zeiss filter set 49 (Excitation 365 nm, Emission 445/50 nm) was used. To analyze permeabilization of *E. coli* and *C. glutamicum* cell membranes by CTAB treatment, the LIVE/DEAD^TM^ BacLight^TM^ Bacterial Viability Kit was used according to the manufacturer’s instructions (Thermo Scientific, Waltham, MA, United States) and the AF microscope filter to analyze propidium iodide staining of the permeabilized cell (excitation at 545 ± 30 nm, emission at 610 ± 75 nm). Cells were cultivated and washed as described above for kinetic fluorescence measurements, CTAB was added to the washed cells at concentrations indicated in results 5 min prior before samples were analyzed by microscopy.

For *in situ* calibration of the sensor signals, cells were harvested and washed with PBS as described above for kinetic fluorescence measurements. The washed cells were transferred to black 96-well plates and 0.05% (w/v) CTAB (final concentration) were added. After 2 min of incubation at room temperature, NADPH at concentrations from 0.01 to 1 mM was added to the CTAB treated cells and subsequently fluorescence was measured at 395 and 451 nm for excitation and emission, respectively. Thereby obtained fluorescence values were plotted against the NADPH concentrations and the resulting calibration curve was then used for the quantification of the intracellular NADPH concentrations in the fluorescence kinetic measurements described above.

### *In vitro* Characterization of mBFP

For purification of mBFP *E. coli* (pCN_*mBFP*) cells were cultivated in TB-medium (Hobbs and Tartoff, 1987) to an OD_600_ of 12. Cells were harvested by centrifugation (20 min, 3,200 ×*g*, 4°C, Eppendorf 5804 R Centrifuge), washed twice with start buffer (50 mM KH_2_PO_4_, pH 7), suspended in start buffer, and disrupted using a Branson Sonifier 250 (Branson Ultrasonics, Danbury, CT, United States). Amplitude was set to 90%, cycle 0.5 and 10 times 30 s burst intervals with intermittent cooling on ice were performed. After removal of cell debris by centrifugation (16,000 ×*g*, 4°C, 15 min, Eppendorf 5804 R Centrifuge) the supernatant was centrifuged at 60,000 ×*g*, 4°C, 1 h (Beckmann XPN 100 ultracentrifuge) to remove the membrane fraction. The cytosolic fraction was diluted 1:3 with start buffer and applied to a HiScreen Capto Blue column (GE Healthcare) equilibrated with start buffer on an Äkta Purifier chromatography system (GE Healthcare). After washing the column with 40 mL start buffer to remove unspecific bound proteins, bound proteins were eluted using a gradient step with elution buffer (1.5 M KCl, 50 mM KH_2_PO_4_, pH 7). Fractions were collected, screened by activity and fluorescence analysis, and analyzed by SDS-PAGE according to Laemmli (1970).

Thermoshift assays were done in a CFX96^TM^ real-time PCR detection system (BioRad Laboratories, Hercules, CA, United States) using SYPRO orange fluorescent dye (Sigma Aldrich, St. Louis, MO, United States) as described (Niesen et al., 2007).

Fluorescence of the purified mBFP in absence and presence of NADPH was determined as described above in an TECAN infinite M200 plate reader at an emission of 451 nm and excitation at 390 nm.

Enzymatic activity of mBFP with different substrates and cofactors was determined using an Ultrospec 2100pro photometer (GE Healthcare). The reaction was performed at 30°C in phosphate buffer (0.4 g/L KH_2_PO_4_, 12.6 g/L K_2_HPO_4_, pH 8). NADPH and NADH were used in concentrations of 200 μM for the determination of benzaldehyde dependent activity (or other substrates, see Supplementary Material). Varying benzaldehyde concentrations were used for the determination of kinetic parameters (*K*_M_, *k*_cat_). Protein concentrations were determined with the Roti-Nanoquant kit (Carl Roth, Karlsruhe, Germany) using a BSA standard.

## Results and Discussion

### The NADPH Sensor Protein mBFP Possesses Benzaldehyde Reductase Activity

**FIGURE 1:**
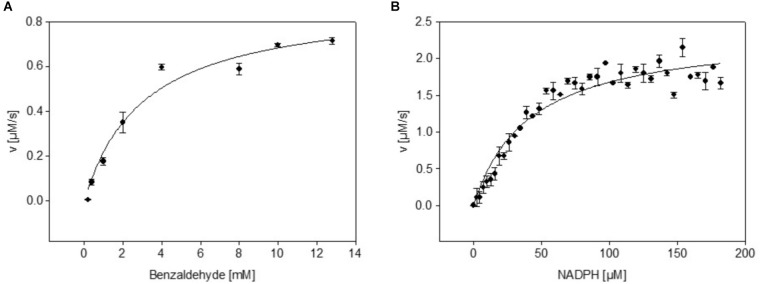
Dependence of dehydrogenase activity of purified mBFP from its substrate benzaldehyde **(A)** and its cofactor NADPH **(B)**; different concentrations of benzaldehyde (0.5–12.5 mM; **(A)** and NADPH (0.5–180 μM; **(B)** were tested in presence of 200 μM NADPH and 12.5 mM benzaldehyde, respectively. Data represent mean values from three independent measurements, data were fitted according to the Michaelis–Menten equation.

Genetically encoded biosensors offer various opportunities for analysis of intracellular metabolites in microorganisms in the course of cultivation and the development of novel strain selection strategies (Eggeling et al., 2015; Cheng et al., 2018). Fluorescent proteins such as mBFP, whose fluorescent properties exclusively rely on the binding of the metabolite of interest, are good candidates for *in vivo* analytics. The metagenome derived blue fluorescent protein mBFP exhibits blue fluorescence upon binding of NADPH independently of the availability of oxygen (Hwang et al., 2012). mBFP was here produced with *E. coli* (pCN_mBFP), which carries the plasmid pCN_mBFP for constitutive high expression of *mBFP* in *E. coli* strains (Spaans et al., 2015). After cell growth, harvest and disruption the mBFP protein was purified from cell free extracts of *E. coli* (pCN_mBFP) to apparent homogeneity by affinity chromatography using a HiScreen Capto Blue column (Supplementary Figure S1). Previous studies on mBFP disregarded the possibility that mBFP may possess an enzymatic function (Polizzi et al., 2007; Buysschaert et al., 2013) albeit it shares high sequence similarities with other, well characterized, enzymatically active SDR (Supplementary Figure S2). We tested purified mBFP with different known substrates of SDRs thereby using either NADPH or NADH as coenzyme. Benzaldehyde in combination with NADPH as cofactor was found to be the preferred substrate of mBFP (Supplementary Table S2). Minor enzyme activities were also detected for mBFP with a series of different other aldehydes as substrates (Supplementary Table S2), however, activities of mBFP with different substrates were detected exclusively with NADPH as a cofactor. Further analysis of mBFP activity with different amounts of the substrate benzaldehyde and the cofactor NADPH revealed saturation kinetics for both compounds. The activity data were fitted to Michaelis–Menten kinetics and resulted in a *K*_M_ value of 3.1 mM of mBFP for benzaldehyde (Figure 1A) and a *k*_cat_ of 4.5 1/s. Analysis of the mBFP activity data with different NADPH concentrations resulted in a *K*_M_ value of 41 μM for NADPH and a *k*_cat_ of 11.85 1/s (Figure 1B). With NADH as co-factor, no enzymatic activity of mBFP was detected. From these results, we conclude that mBFP is an active reductase with a preference for NADPH as cofactor.

### mBFP Exclusively Binds NADPH

**FIGURE 2:**
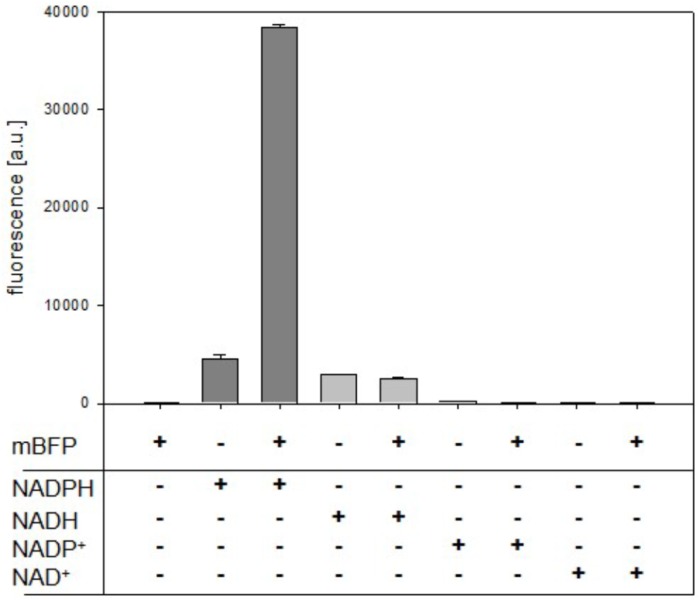
Effects of purified mBFP on fluorescence of NADPH, NADH, NADP^+^, and NAD^+^ (each at 0.5 mM) at excitation with 395 nm and emission at 451 nm. Data represent mean values and SDs of three independent measurements.

The quality of a metabolite sensor stands or falls with the specificity of the signal induced by the detected molecule. Noticeable, mBFP amplifies the intrinsic fluorescence of NADPH upon binding as it is described for other SDRs (Polizzi et al., 2007; Buysschaert et al., 2012). As expected purified mBFP apoenzyme shows no emission at 451 nm when excited at 395 nm, but its presence lead to an increase of the emission signal for 0.5 mM NADPH about 8.5 fold from 4,532 ± 408 (pure NADPH) to 38,402 ± 277 (NADPH + mBFP) (Figure 2). For NADH no amplification of its intrinsic fluorescence by addition of mBFP was detected (Figure 2), demonstrating the specificity of the mBFP for NADPH.

**FIGURE 3:**
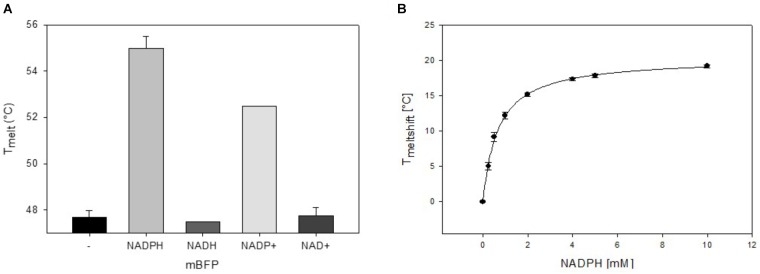
Thermal shift assays for the analysis of the cofactor preference of mBFP. Melting temperatures of apo-mBFP without (–) or in the presence of (1 mM each) NADPH, NADH, NADP^+^, or NAD^+^ **(A)**. Dependence of mBFP melting temperature on the concentration of its cofactor NADPH **(B)**, NADPH concentrations of 0–10 mM were tested. Melting temperatures were determined by heating from 40 to 70°C in 0.5°C steps, and unfolding was monitored as described in Section “Materials and Methods.” Data represent mean values and SDs of three independent measurements, data in **(B)** were fitted according to the Michaelis–Menten equation.

To analyze mBFPs affinity for different cofactors, thermal degradation assays (thermofluor) were performed. In these experiments binding of a cofactor leads to a shift in melting temperature (*T*_Melt_) of the protein under investigation (Niesen et al., 2007). The *T*_Melt_ of 47.5°C determined for the mBFP apoenzyme was shifted to 55°C upon addition of 1 mM NADPH and 52°C upon addition of 1 mM NADP^+^ (Figure 3A and Supplementary Figure S3A), which implies a higher affinity of mBFP toward NADPH than NADP^+^. Binding of mBFP to NADPH and NADP is plausible as redox enzymes often catalyze reversible reactions. For further characterization, effects of different NADPH concentrations (0–10 mM) on *T*_Melt_ of mBFP were analyzed (Figure 3B). Non-linear regression of the resulting data revealed a saturation kinetics with a dissociation constant (*K*_D_) of 0.64 mM (Figure 3B and Supplementary Figure S3B). Interestingly only presence of phosphorylated cofactors led to an increase in *T*_Melt_ of mBFP, whereas *T*_Melt_ determined in presence of the non-phosphorylated cofactors NADH (47°C) and NAD^+^ (47.5°C) were identical to *T*_Melt_ of the apoenzyme (Figure 3A). This result indicates that mBFP does not bind to NADH and NAD^+^. The broad dynamic range of fluorescence amplification in combination with the high specificity for NADPH makes mBFP a suitable candidate to be used as NADPH biosensor for *in vivo* applications.

### Use of the Genetically Encoded NADPH Sensor mBFP in *C. glutamicum*

Large-scale microbial production of amino acids such as L-lysine heavily relies on efficient recycling of the cofactor NADPH (Eggeling and Bott, 2015; Xu et al., 2018), which has therefore been a well-studied target for the engineering of *C. glutamicum* strains (Blombach and Seibold, 2010; Wang et al., 2016). For analysis of NADPH levels in *C. glutamicum* via mBFP as a genetically encoded sensor, the plasmid pEKEx2_mBFPopt was constructed. For this purpose, an mBFP gene was synthesized with codon-optimization for *C. glutamicum* and cloned into the plasmid pEKEx2. The sensor plasmid was introduced in *C. glutamicum* WT and the phosphoglucoisomerase-deficient mutant strain *C. glutamicum* Δ*pgi*. Deletion of *pgi* results in the redirection of the carbon flux from glycolysis to the pentose-phosphate pathway, which brings about the generation of high NADPH concentrations in this strain (Marx et al., 2003). To analyze NADPH concentrations with the sensor mBFP, cells of *C. glutamicum* WT (pEKEx2_mBFPopt) and *C. glutamicum* Δ*pgi* (pEKEx2_mBFPopt) were cultivated in CgXII medium with 100 mM glucose as substrate, sampled after 4 h of cultivation, and their fluorescence analyzed in a plate reader. A significantly higher fluorescence of 2,125 ± 551 FLU/OD at excitation 395 nm with and emission at 451 nm was detected for *C. glutamicum* Δ*pgi* (pEKEx2_mBFPopt) when compared to *C. glutamicum* (pEKEx2_mBFPopt), for which a fluorescence of 613 ± 167 FLU/OD was determined.

Analyses of these cells by fluorescence microscopy revealed besides the overall increased fluorescence of *C. glutamicum* Δ*pgi* (pEKEx2_mBFPopt) when compared to *C. glutamicum* (pEKEx2_mBFPopt) that the fluorescence showed only minor cell–cell variations for each strain (Supplementary Figure S4). As for *C. glutamicum* Δ*pgi* an increased NADPH concentration is expected during cultivation with glucose when compared to *C. glutamicum* WT, the observed higher overall fluorescence for *C. glutamicum* Δ*pgi* (pEKEx2_mBFPopt) indicates that by the use of mBFP NADPH levels can be monitored in *C. glutamicum*.

### mBFP Enables Detection of Fast Alterations of NADPH Concentrations in *C. glutamicum* and *E. coli*

Current techniques for analyses of intracellular NADPH concentrations are mostly employed to study steady state levels but have a limited capacity for analyses of dynamic changes. However, the latter are the key for the understanding of sensing and adaptive mechanisms, drug modes of action, and homeostasis mechanisms (Zampieri et al., 2017). In bacteria, levels of metabolites often change upon perturbations within seconds (Chubukov et al., 2014; Wegner et al., 2015). These fast changes cannot be observed in real-time by the use of genetically encoded sensors requiring transcription and translation of a fluorescent reporter, but the properties of protein based sensors are matching this task (Liu et al., 2015; Lin et al., 2017). The protein sensor mBFP binds and enhances specifically the fluorescence of NADPH and was above shown to be suited for measurements of intracellular NADPH concentrations in *C. glutamicum*.

**FIGURE 4:**
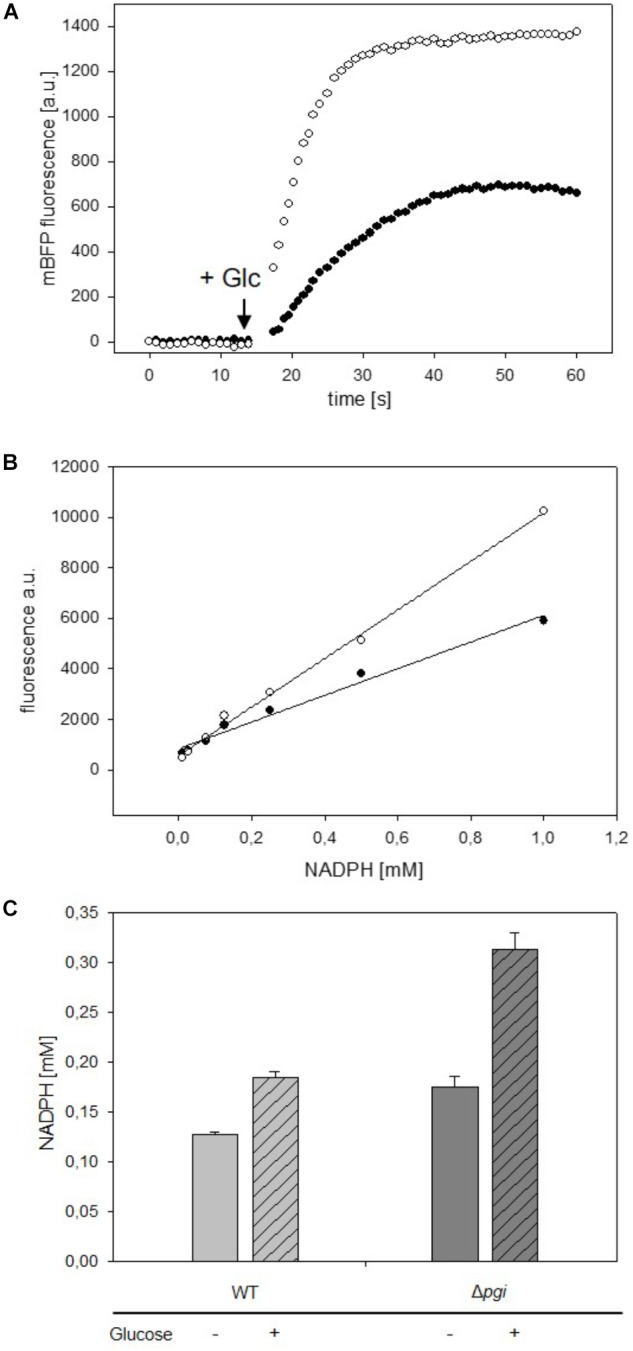
Analyses of changes of mBFP fluorescence in starved cells of *C. glutamicum* WT (pEKEx2-mBFPopt) [filled circles] and *C. glutamicum* Δ*pgi* (pEKEx2-mBFPopt) [open circles] upon addition of the substrate glucose (indicated by the arrow) **(A)**. *In situ* calibration of mBFP derived signals by the use CTAB permeabilized cells from *C. glutamicum* WT (pEKEx2-mBFPopt) [filled circles] and *C. glutamicum* Δ*pgi* (pEKEx2-mBFPopt) [open circles] **(B)** in presence of different amounts of added NADPH. Steady state levels of intracellular NADPH concentrations calculated based on *in situ* calibrations in *C. glutamicum* WT (pEKEx2-mBFPopt) and *C. glutamicum* Δ*pgi* (pEKEx2-mBFPopt) before and after glucose addition **(C)**. For panels **(A,B)** one representative experiment of a series of three independent experiments is shown. Data in **(C)** represent mean values and SDs of three independent experiments.

To test utilization of mBFP for the detection of fast changes of NADPH levels, the response to fast changes of nutrient availability was analyzed in *C. glutamicum* strains carrying mBFP. *C. glutamicum* WT (pEKEx2_mBFPopt) and *C. glutamicum* Δ*pgi* (pEKEx2_mBFPopt) were cultivated for 12 h in 2xTY complex medium with 1 mM IPTG to induce synthesis of mBFP. The derived nutrient-starved cells of *C. glutamicum* WT (pEKEx2_mBFPopt) and *C. glutamicum* Δ*pgi* (pEKEx2_mBFPopt) were washed twice with PBS, suspended in PBS and transferred into single wells of a 96-well plate. Kinetic assays were performed in a Tecan M200 plate reader equipped with an injector module: mBFP fluorescence was read in 2 s intervals for 2 min and glucose was injected automatically into the wells after the first 15 s of the kinetic assay. As depicted in Figure 4A, a very fast increase of the mBFP fluorescence within 30–40 s after the addition of glucose to 658 FLU and 1,374 FLU can be seen for *C. glutamicum* WT (pEKEx2_mBFPopt) and *C. glutamicum* Δ*pgi* (pEKEx2_mBFPopt), respectively. As the addition of glucose and its metabolization via glycolysis and tricarboxylic acid cycle besides NADPH probably also causes formation of the redox intermediates NADH and FADH (Blombach and Seibold, 2010), which both also show fluorescence at an emission wavelength of 451 nm when excited at 390 nm, changes of fluorescence upon glucose addition were also analyzed for cells of the control strains *C. glutamicum* WT (pEKEx2) and *C. glutamicum* Δ*pgi* (pEKEx2). As depicted in Supplementary Figure S5 the fluorescence increased after the addition of glucose from initially 14 FLU to 19 FLU and from 10 FLU to 50 FLU in *C. glutamicum* WT (pEKEx2) and *C. glutamicum* Δ*pgi* (pEKEx2), respectively. As fluorescence values obtained for the two mBFP carrying strains *C. glutamicum* WT (pEKEx2_mBFPopt) and *C. glutamicum* Δ*pgi* (pEKEx2_mBFPopt) are about 30 times higher than the fluorescence values measured for the mBFP-deficient control strain, the amplification of NADPH fluorescence by mBFP effectively masks the fluorescence derived from other redox intermediates such as NADH and FADH in the mBFP carrying strains.

For determination of intracellular NADPH concentrations we calibrated the sensor *in situ* in cells of *C. glutamicum* WT (pEKEx2_mBFPopt) and *C. glutamicum* Δ*pgi* (pEKEx2_mBFPopt), which were permeabilized by addition of 0.05% (w/v) of CTAB (final concentration). At this CTAB concentration small pores are formed in the membrane, which allow the fast diffusion of small molecules, however, the cell’s superstructure remains intact (Supplementary Figure S6). As depicted in Figure 4B, different slopes were observed for the linear regression lines based NADPH dependent mBFP fluorescence of permeabilized cells of *C. glutamicum* WT (pEKEx2_mBFPopt) and *C. glutamicum* Δ*pgi* (pEKEx2_mBFPopt), which is probably brought about different levels of mBFP formed in course of the pre-cultivation. Based on the *in situ* calibration for *C. glutamicum* WT (pEKEx2_mBFPopt) an increase of the NADPH concentration from initially 0.127 to 0.185 mM after the addition of glucose was determined (Figure 4C). The initial NADPH concentration of 0.175 mM determined for starved cells of *C. glutamicum* Δ*pgi* (pEKEx2_mBFPopt) is higher than the initial concentration determined for *C. glutamicum* WT (pEKEx2_mBFPopt), and after glucose addition the NADPH concentration in *C. glutamicum* Δ*pgi* (pEKEx2_mBFPopt) increased up to 0.313 mM. The intracellular concentrations already reached within 1 min after the addition of the substrate in both *C. glutamicum* WT (pEKEx2_mBFPopt) and *C. glutamicum* Δ*pgi* (pEKEx2_mBFPopt) are in the same concentration range as the NADPH concentrations determined via metabolite extraction and subsequent HPLC-MS analyses for WT and Pgi-deficient *C. glutamicum* cells, respectively, in previous studies (Marx et al., 2003; Bartek et al., 2010).

**FIGURE 5:**
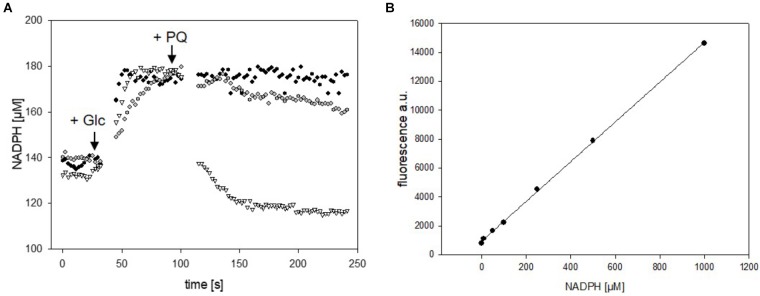
Analyses of changes of NADPH concentrations in cells of *E. coli* DH5α (pEKEx2_mBFPopt) upon addition of the substrate glucose (indicated by the arrow, 100 mM) and consecutive addition of different amounts of paraquat (8 mM paraquat—gray circles, 16 mM paraquat—white triangles, no addition of paraquat—black circles) **(A)**. *In situ* calibration of mBFP derived signals by the use CTAB permeabilized cells from *E. coli* DH (pEKEx2_mBFPopt) in presence of different amounts of added NADPH **(B)**. One representative experiment of a series of three independent experiments is shown.

Versatility of the mBFP-based method for observation of fast variations of NADPH concentrations was also tested in *E. coli* DH5α (pEKEx2_mBFPopt). For this purpose, cells of *E. coli* DH5α (pEKEx2_mBFPopt) cultivated in 2xTY medium and induced with 1 mM IPTG for 12 h were harvested by centrifugation and carefully washed with PBS. One aliquot of the washed *E. coli* DH5α (pEKEx2_mBFPopt) cells was permeabilized by addition of 0.05% CTAB (Supplementary Figure S7) and then used for an *in situ* calibration of the mBFP sensor signal response to different NADPH concentrations (Figure 5B). Further aliquots of *E. coli* DH5α (pEKEx2_mBFPopt) were transferred to wells of a 96-well plate and used for kinetic assays. Within 30 s upon addition of 100 mM glucose (final concentration) the intracellular NADPH concentration increased in *E. coli* DH5α (pEKEx2_mBFPopt) from 140 to 180 μM, which shows that in both *E. coli* and *C. glutamicum* NADPH accumulates fast in response to increases of nutrient availability. Protein based sensors like mBFP should also enable the observation of fast decreases in their analytes concentrations. Addition of paraquat initiates antioxidative mechanisms in *E. coli*, which lead to the depletion of the NADPH pool (Gu and Imlay, 2011). Indeed, upon addition of paraquat to glucose-fed cells of *E. coli* DH5α (pEKEx2_mBFPopt) the intracellular NADPH concentrations decreased in dependence of the added paraquat concentration (Figure 5A). As expected addition of water did not lead to a change of the intracellular NADPH concentration in control experiments (Figure 5A). These results demonstrate the functionality of mBFP in two different hosts and show that it is highly suitable for the fast dynamic measurements of intracellular NADPH concentrations required to understand redox mechanisms of cells.

## Conclusion

mBFP was here shown to specifically bind to and amplify fluorescence of NADPH, which are essential prerequisites for its use as a genetically encoded biosensor for the analysis of intracellular NADPH concentrations. Plasmid encoded mBFP allowed to measure fast changes of NADPH concentrations in both *C. glutamicum* and *E. coli* strains and most importantly it allowed to analyze increases as well as decreases of NADPH concentrations in real time. It has to be denoted, that each use of mBFP requires calibration of the signals by permeabilization of the cell and incubation with different NADPH concentrations as the mBFP derived fluorescence signal depends both on the internal NADPH concentration as well as the amount of mBFP, which also varies. For short time intervals variations of mBFP amounts can be neglected but to monitor intracellular NADPH levels in the course of fermentations regular sampling to perform calibrations with permeabilized cells is probably necessary. The development of a ratiometric sensor based on mBFP will probably allow to easily monitor NADPH levels also during longtime experiments. The monitoring of NADPH levels in *C. glutamicum* and *E. coli* during cultivations is interesting as NADPH is the redox cofactor limiting production of bulk chemicals synthesized via anabolic pathways such as amino acids (Hirasawa and Shimizu, 2016; Xu et al., 2018), but for this purpose conventional, sampling dependent techniques can be easily employed (Liu et al., 2018). The general advantage of a genetically encoded metabolite sensors for NADPH like mBFP is the site specific analyses of metabolite concentrations, e.g., in to monitor compartment-specific transient changes of intracellular balances of NADPH in eukaryotic cells (Ying, 2008; Wiederkehr and Demaurex, 2017). These measurements are currently performed via analyses of autofluorescence (Blacker et al., 2012; Blacker and Duchen, 2016), which unfortunately coincidences in the case of NADH and NADPH. The specificity of mBFP for NADPH over NADH, the masking of NADH fluorescence by the strong amplification of the NADPH fluorescence signal, and the possibility to monitor fast changes without sampling indicate that mBFP might be a suitable molecular tool for cell biologist. In its present state, the sensor protein mBFP is already a well suited to observe fast changes of intracellular NADPH concentrations in bacteria, which provides a new insight into the kinetics of microbial metabolism.

## Author Contributions

GS and OG designed and coordinated the study. OG and AE carried out the experiments. GS, AE, and OG analyzed the results and wrote the manuscript.

## Conflict of Interest Statement

The authors declare that the research was conducted in the absence of any commercial or financial relationships that could be construed as a potential conflict of interest.

## Abbreviations

CTAB: cetyl trimethylammonium bromide
IPTG: isopropyl β-D-1-thiogalactopyranoside
TF: transcription factor
