# Supplementary material for: Real Time Monitoring of NADPH Concentrations in Corynebacterium glutamicum and Escherichia coli via the Genetically Encoded Sensor mBFP
Source: Front Microbiol. 2018 Oct 24;9:2564. doi: 10.3389/fmicb.2018.02564 (PMC6207642; doi:10.3389/fmicb.2018.02564)
Supplement: Supplementary file 1 [file Data_Sheet_1.PDF]

Supplementary data to the manuscript:

Real time monitoring of NADPH concentrations in *Corynebacterium glutamicum* and *Escherichia coli* via the genetically encoded sensor mBFP

By Oliver Goldbeck, Alexander W. Eck, and Gerd M. Seibold

The supplementary data comprise 7 figures (Fig. S1, S2, S3, S4, S5, S6, S7) and 2 tables (Tab. S1, S2).

Correspondence: Gerd M. Seibold, Institute of Microbiology & Biotechnology, University of Ulm, 89081 Ulm, Germany. Tel.: +49 (0)731 502 4853; fax: +49 (0)731 502 2719; e-mail: gerd.seibold@uni-ulm.de

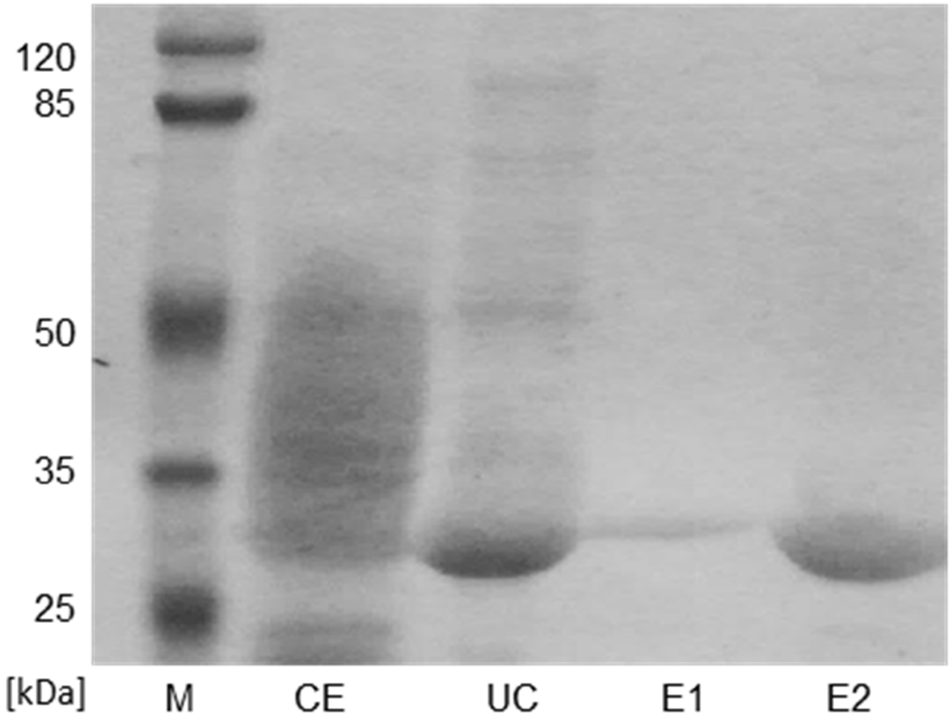

Fig. S1: Purification of recombinant mBFP from E. coli BL21(DE3)(pCN_mBFP) via affinity chromatography using a HiScreen Capto Blue column, SDS-PAGE analysis of each step of the purification procedure: crude cell extract (CE), ultracentrifugation (UC), elution fraction 1 (E1) and 2 (E2) from HiScreen Capto blue column, and PAGE ruler prestained protein ladder (Thermo Scientific) containing proteins of indicated molecular mass.

```
SDRvv       MMGSDKTVYVVLGGTSGIGAELAKQLESEHTIVHVASRQTG-------------------
mBFP        -MQNLNGKVAVTGGSRGIGAAIVRRLAADGADIAFTYVSASSKNVATALVQELEAKG-RR
BFPvv       ----MKKLVVITGASSGIGEAIARRFSEEGHPLLLLARRVE----------RLEALNLPN
                 :   .: *.: ***  :.:::  :   : .    .

SDRvv       -----LDISDEKS----VYHYFETIGAFDHLIVTAGSYAPAGKVVDVEVTQAKYAFDTKF
mBFP        ARAIQADSADPAQVRQAVEQAIVQLGPVDVLVNNAGIF-LAGPLGEVTLDDYERTMNINV
BFPvv       TLCAQVDVTDKNTFDAAITRAEKIYGPADVLVNNAGVM-LLGQIDTQEANEWQRMFDVNV
                  * :*       : :     *  * *: .**     * :      : :  :: :.

SDRvv       WGAVLAAKHGA--RYLKQGGSITLTSGMLSRKV-VANTYVKAAINAAIEATTKVLAKEL-
mBFP        RAPFVAIQAAQASM--PDGGRIINIGSCLAERAGRAGVTLYAASKSALLGMTRGLARDLG
BFPvv       LGLLNGMQAVLAPMKARNSGTIINISSIAGKKT-FPDHAAYCGTKFAVHAISENVREEVA
             . . . :         :.* *   ..  ..:.   .    .. : *: . :. : .::

SDRvv       -APIRVNAISPGLTKTEAYKGMNADDRDAMYQRTQSHLPVGKVGEASDIAMAYLFAI---
mBFP        ARGITANVVHPGPIDTDMNPADGERS-----GELVAVLSLPHYGEVRDIAGMVAFL-AGP
BFPvv       ASNVRVTTIAPGAVETELLSHTTSQQI--KDGYDAWKVDMGGVLAADDVARAVLFAYQQP
               : ...: **  .*:        .           : :     . *:*    *

SDRvv       QNSYMTGTVIDVDGGALLG
mBFP        DGRYVTGASLAVDGGFAA-
BFPvv       QNVCIREIALAPTKQQP--
            :.  :    :
```

Fig. S2: Alignment of the amino acid sequences of mBFP (sequence published by Hwang et al. 2012), the short-chain dehydrogenase SDRvv from *Vibrio vulnificus* (Accesion number ASC60033.1; Buysschaert et al. 2013), and the blue fluorescent protein BFPvv from *Vibrio vulnificus* (Accesion number AAO08298.1; Su et al. 2001). Asterisk (*) indicates positions which have a single, fully conserved residue. Colon (:) indicates conservation between groups of strongly similar properties and period (.) indicates conservation between groups of weakly similar properties. Residues displayed in red are part of the conserved GxxGxG nucleotide binding motif. Residues displayed in blue are conserved residues of a catalytic tetrade presumably involved in oxidoreductase activity. For the alignment ClustalW was used as described by Larkin et al. 2007.

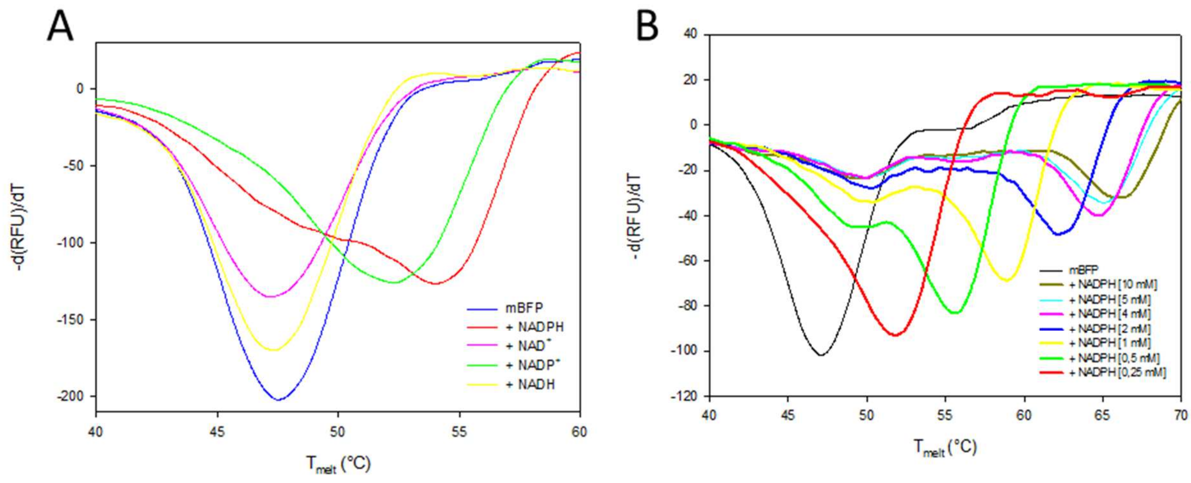

Fig. S3: Thermal shift assays for the analysis of the cofactor preference of mBFP. Melting temperatures of apo-mBFP without (blue) or in the presence of (10 mM each) NADPH (red), NADH (yellow), $NADP^+$ (green), or NAD^+ (pink) [A]. Dependence of mBFP melting temperature on the concentration of its cofactor NADPH [B], NADPH concentrations of 0 to 10 mM were tested. Melting temperatures were determined by heating from 40 to 70 °C in 0.5 °C steps, and unfolding was monitored as described in Materials and methods.

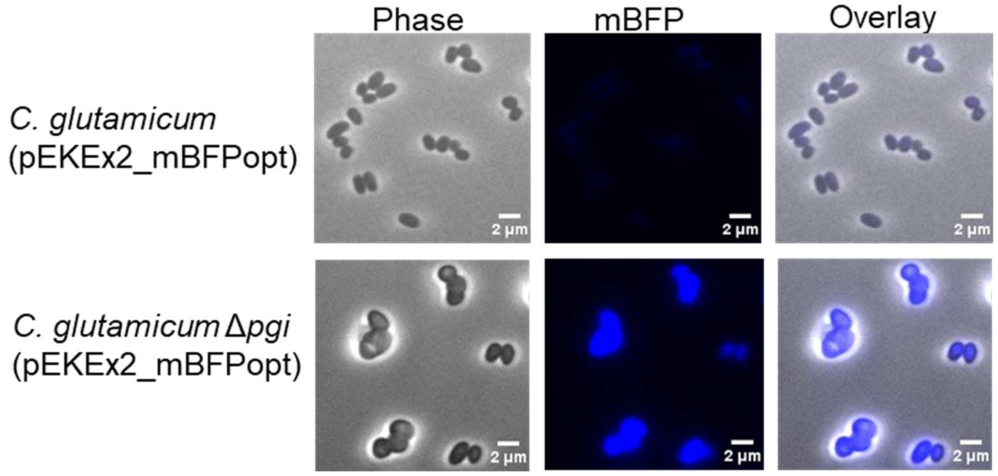

Fig. S4: Microscopic analysis of mBFP fluorescence in cells of *C. glutamicum* WT (pEKEx2-mBFPopt) and *C. glutamicum* Δ*pgi* (pEKEx2-mBFPopt) cultivated in presence of glucose. Shown are phase-contrast images, fluorescence analyses (excitation at 365 nm and emission at 445/50 nm), and overlays. Cells were cultivated in CgXII medium with 100 mM glucose, washed twice with PBS, suspended in PBS, and incubated in presence of 100 mM glucose for 10 min before mounting on an agarose pad and microscopic analyses.

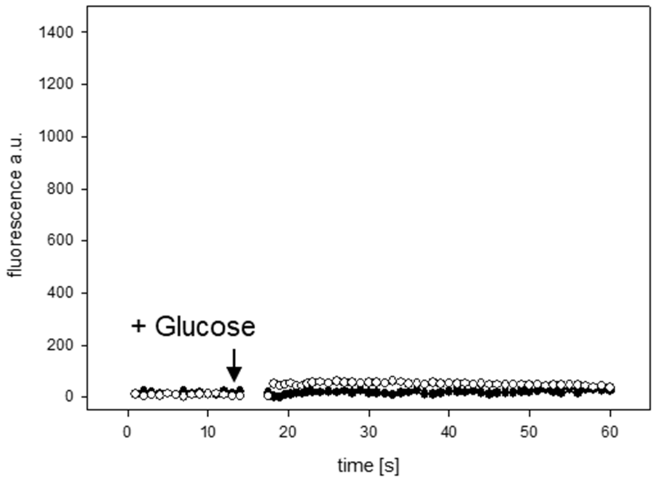

Fig. S5: Analyses of changes of fluorescence in starved cells of *C. glutamicum* WT (pEKEx2) [filled circles] and *C. glutamicum* Δ*pgi* (pEKEx2) [open circles] upon addition of the substrate glucose (indicated by the arrow) upon excitation at 395 nm and emission at 451nm. One representative experiment of a series of three independent experiments is shown.

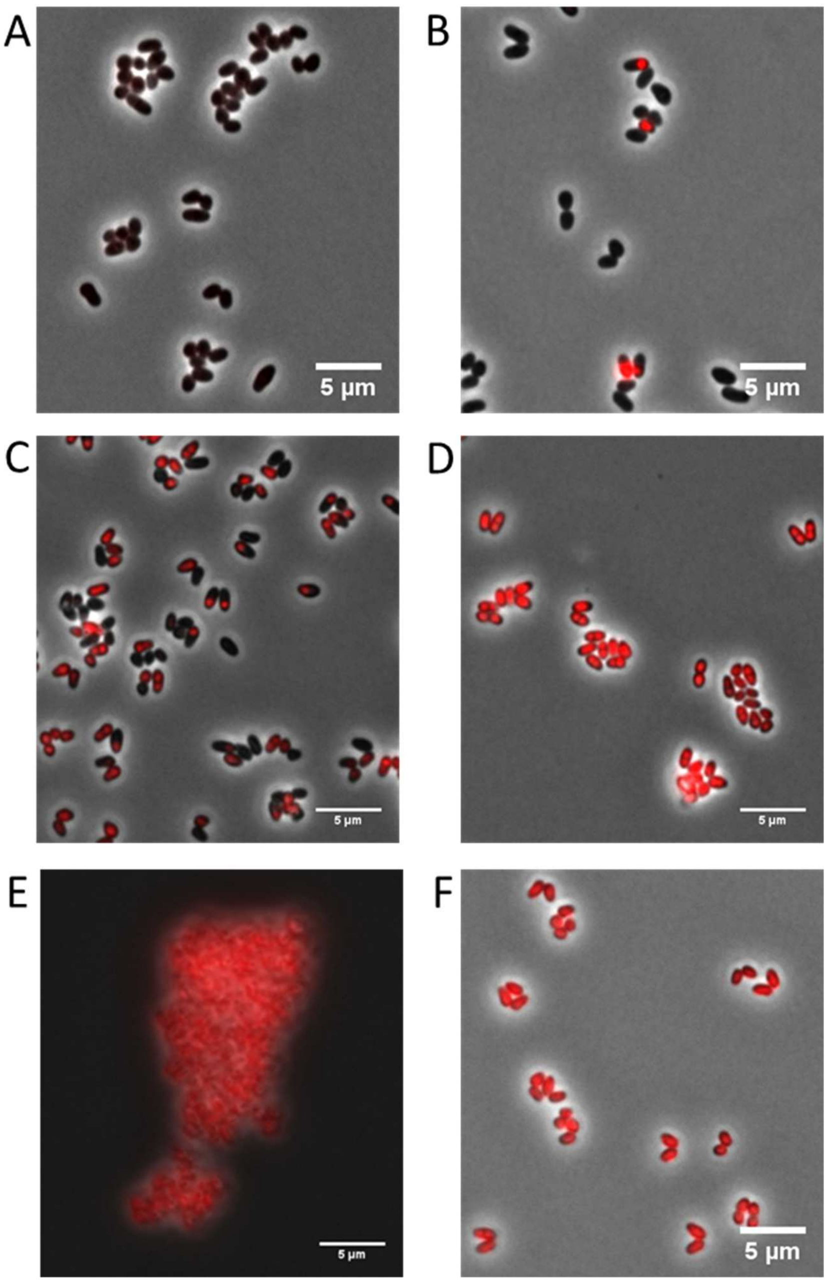

Fig. S6: Analyses of permeabilisation of *C. glutamicum* cells with different amounts of CTAB by the use of the membrane impermeable dye propidium iodide. Cells were untreated (A, negative control), treated with different amounts of CTAB (0.005% B, 0.01% C, 0.05% D, 0.1 % E) or 30% isopropanol (positive control), stained with propidium iodide, and then analysed by fluorescence microscopy.

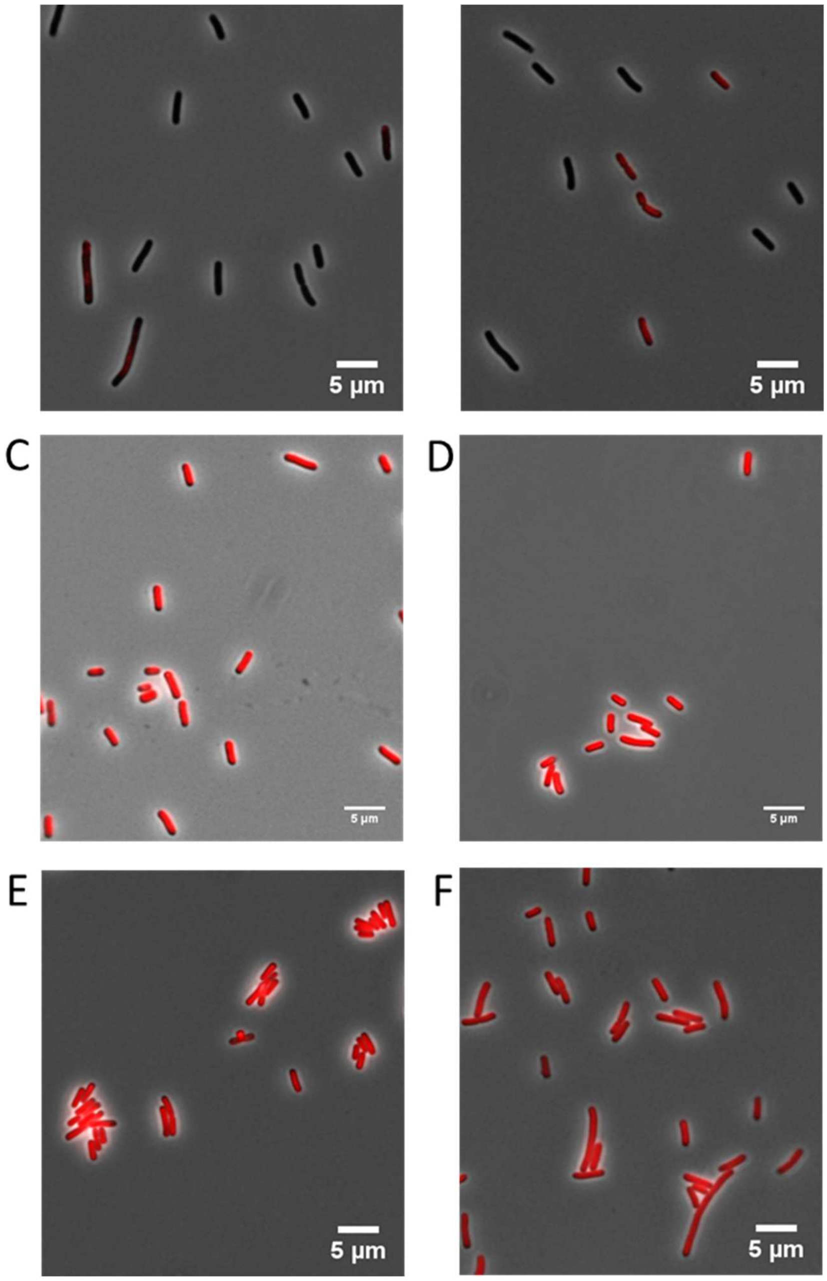

Fig. S7: Analyses of permeabilisation of *E. coli* cells with different amounts of CTAB by the use of the membrane impermeable dye propidium iodide. Cells were untreated (A, negative control), treated with different amounts of CTAB (0.005% B, 0.01% C, 0.05% D, 0.1 % E) or 30% isopropanol (positive control), stained with propidium iodide, and then analysed by fluorescence microscopy.

Table S1: Oligonucleotides used in this study

Oligonucleotide	Sequence (3´- ´5)	Restriction sites[1]
mBFPopt_fw	CAGCAGGTCGACAGGAGTATTATGCAGAATCTGAACGGC	*Sal*I
mBFPopt_rev	GATCAGGAGCTCTTAAGCGGCGAAGCCGCCGTC	*Sac*I
mBFPopt;<br>Genfragment<br>optimized for<br>*C. glutamicum*	ATGCAGAATCTGAACGGCAAAGTGGCTTTCGTGACCGGCGGCAGCCGCG<br>GCATCGGCGCGGCGATCGTCCGCCGCTTGGCGGCGGACGGCGCCGACAT<br>CGCGTTCACCTATGTCAGCGCCTCGTCGAAAAACGTGGCCACCGCCCTGG<br>TGCAAGAACTCGAGGCCAAGGGCCGCCGCGCTCGCGCCATCCAGGCGGA<br>CTCGGCGGATCCGGCCCAGGTGCGGCAGGCGGTCGAGCAGGCCATCGTG<br>CAACTGGGGCCGGTGGACGTGCTGGTGAACAACGCCGGCATCTTCCTGG<br>CCGGCCCCTTGGGCGAGGTGACGCTGGACGACTACGAACGCACGATGAA<br>CATCAATGTGCGCGCGCCTTTCGTGGCCATCCAGGCCGCGCAGGCCTCGA<br>TGCCGGACGGCGGCCGCATCATCAACATCGGCAGCTGCCTGGCGGAACG<br>CGCCGGCCGAGCCGGGGTAACGCTGTATGCCGCCAGCAAGTCGGCGCTG<br>CTGGGCATGACGCGCGGCCTGGCGCGCGACCTGGGCGCGCGCGGCATCA<br>CCGCCAACGTCGTGCACCCGGGCCCGATCGACACCGACATGAATCCCGC<br>AGATGGCGAACGCTCGGGCGAACTGGTGGCCGTGCTGTCCTTGCCTCATT<br>ACGGCGAGGTGCGCGACATCGCCGGCATGGTGGCTTTCCTGGCCGGGCC<br>GGATGGGCGCTACGTGACCGGTGCGAGTCTGGCGGTGGACGGCGGCTTC<br>GCCGCTTAA	

[1] restriction sites are underlined

Table S2. Analyses of mBFP oxidoreductase activity with different substrates and NADPH and NADH as cofactor. Data represent mean values and standard deviations of at least three independent experiments.

Substrate	Activity with NADPH (U/mg)	Activity with NADH (U/mg)
Benzaldehyde	0.902 ± 0.02	n.d.
Acetaldehyde	n.d.	n.d.
Vanillin	n.d.	n.d.
p-Anisaldehyde	0.185 ± 0.01	n.d.
Acetylacetone	0.045 ± 0.01	n.d.
Acetone	n.d.	n.d.
1-butanol	n.d.	n.d.

n.d. = not detected

References cited in supplementary data

Buysschaert G, Verstraete K, Savvides SN, Vergauwen B. 2013. Structural and biochemical characterization of an atypical short-chain dehydrogenase/reductase reveals an unusual cofactor preference. FEBS J 280(5):1358-70.

Hwang CS, Choi ES, Han SS, Kim GJ. 2012. Screening of a highly soluble and oxygen-independent blue fluorescent protein from metagenome. Biochem Biophys Res Commun 419(4):676-81.

Larkin MA, Blackshields G, Brown N, Chenna R, McGettigan PA, McWilliam H, Valentin F, Wallace IM, Wilm A, Lopez R. 2007. Clustal W and Clustal X version 2.0. bioinformatics 23(21):2947-2948.

Su J-H, Chuang Y-C, Tsai Y-C, Chang M-C. 2001. Cloning and characterization of a blue fluorescent protein from *Vibrio vulnificus*. Biochemical and biophysical research communications 287(2):359-365.
